# Reducing Motor Vehicle-Related Injuries at an Arizona Indian Reservation: Ten Years of Application of Evidence-Based Strategies

**DOI:** 10.9745/GHSP-D-15-00249

**Published:** 2015-12-15

**Authors:** Stephen R Piontkowski, Jon S Peabody, Christine Reede, José Velascosoltero, Gordon Tsatoke, Timothy Shelhamer, Kenny R Hicks

**Affiliations:** ^a^​ Phoenix Area Indian Health Service, Lakeside, AZ, USA; ^b^​ San Carlos Police Department, San Carlos, AZ, USA; ^c^​ California Area Indian Health Service, Sacramento, CA, USA

## Abstract

Unintentional injury is a significant public health burden for American Indians and Alaska Natives and was the leading cause of death among those aged 1 to 44 years between 1999 and 2004. Of those deaths, motor vehicle-related deaths cause the most mortality, justifying the need for intervention at an American Indian Reservation in Arizona (United States). We describe motor vehicle injury prevention program operations from 2004 through 2013. This community-based approach led by a multidisciplinary team primarily comprised of environmental public health and law enforcement personnel implemented evidence-based strategies to reduce the impact of motor vehicle-related injuries and deaths, focusing on reducing impaired driving and increasing occupant restraint use. Strategies included: mass media campaigns to enhance awareness and outreach; high-visibility sobriety checkpoints; passing and enforcing 0.08% blood alcohol concentration limits for drivers and primary occupant restraint laws; and child car seat distribution and education. Routine monitoring and evaluation data showed a significant 5% to 7% annual reduction of motor vehicle crashes (MVCs), nighttime MVCs, MVCs with injuries/fatalities, and nighttime MVCs with injuries/fatalities between 2004 and 2013, but the annual percent change in arrests for driving under the influence (DUI) was not significant. There was also a 144% increase in driver/front seat passenger seat belt use, from 19% in 2011 before the primary occupant restraint law was enacted to 47% during the first full year of enforcement (2013). Car seat checkpoint data also suggested a 160% increase in car seat use, from less than 20% to 52% in 2013. Implementation of evidence-based strategies in injury prevention, along with employment of key program approaches such as strong partnership building, community engagement, and consistent staffing and funding, can narrow the public health disparity gap experienced among American Indian and Alaska Native communities.

## Abstract (precis)

Motor vehicle crashes decreased and seat belt use, including car seat use, increased in an American Indian and Alaska Native community through a multidisciplinary approach using strong partnerships among public health and law enforcement agencies; community outreach; mass media campaigns; and enactment and high-visibility enforcement of key laws, such as lowering the legal blood alcohol concentration limit for drivers and mandating use of occupant restraints.

## INTRODUCTION

Between 1999 and 2004, unintentional injury was the leading cause of death among American Indians and Alaska Natives (AI/AN) ages 1 to 44 years in the United States.^1^ The impact of unintentional injury was even more acute for AI/AN in Arizona, for whom it was the leading cause of death for all ages during the same time period.^1^ The leading cause of unintentional injury death for AI/AN in Arizona was motor vehicle-traffic related, which accounted for 60.5% of all unintentional injury deaths.^1^ Nationally in 2005, the age-adjusted motor vehicle-related death rate among AI/AN was 2.01 times higher than among all races.^2^

Unintentional injury is a leading cause of death among American Indians and Alaska Natives in the United States.

Injuries—whether fatal or non-fatal—impose obvious impacts in AI/AN populations on the affected victims, families, and communities. However, injury can also have less obvious impacts. Due to the higher incidence of injury in younger age groups, fatal injury may involve premature death. Years of Potential Life Lost (YPLL) measures premature death before age 65 to illustrate lost potential productivity and income. Unintentional injury is the leading YPLL cause of death category for AI/AN, accounting for 28% of all YPLL causes of death, compared with 8% each for the next leading causes of heart disease and cancer.^3^ Injuries also pose an enormous economic burden among AI/AN populations. The lifetime costs of all injuries among AI/AN populations nationally in 2000 was estimated at over 2 billion dollars.^3^

The Indian Health Service (IHS) has long recognized the problem that injury presents in AI/AN populations and has focused attention and resources on injury prevention activities. The US Centers for Disease Control and Prevention (CDC) has similarly recognized the injury problem in AI/AN populations and has collaborated with the IHS and with individual tribes. However, until relatively recently funding to support tribes to develop their own local infrastructures to address local injury problems based on the individual tribe’s needs was rare.

A focus of both the IHS Injury Prevention Program and the CDC’s National Center for Injury Prevention and Control is the implementation of effective injury prevention strategies that are evidence-based or that are considered best practices. Among AI/AN populations, appropriate attention has been focused on the prevention of motor vehicle-related injury. Risk factors for motor vehicle crash-related injuries and deaths in AI/AN communities include low rates of occupant restraint use and a high prevalence of alcohol-impaired driving.^4^ Accordingly, recommended effective strategies to prevent or reduce motor vehicle-related injury associated with low occupant restraint use and impaired driving include mandatory occupant restraint (child safety seats and seat belts) laws, enhanced enforcement campaigns such as occupant restraint checkpoints, lowering legal blood alcohol concentration (BAC) limits for drivers from 0.10% to 0.08%, sobriety checkpoints, and community-wide messaging about occupant restraint laws or mass media campaigns about alcohol-impaired driving.^5^^,^^6^ The “Guide to Community Preventive Services”^5^ is a systematic review of community-based interventions managed by the CDC. The systematic review process assesses large bodies of scientific literature by applying the scientific process to reduce bias in how conclusions are reached, improves the power and precision of results, and summarizes evidence about the effectiveness of specific interventions. This evaluation leads to recommended evidence-based interventions that have the greatest public health impact, allowing public health programs to focus on what works, rather than on determining, or guessing, what might work.

Risk factors for motor vehicle-related injuries include low rates of occupant restraint use and high prevalence of alcohol-impaired driving.

In 2004, the San Carlos Apache Tribe in Arizona received funding from the CDC to develop, implement, and evaluate a multiyear program using such effective strategies to combat impaired driving. Administratively located within the San Carlos Police Department, this Motor Vehicle Injury Prevention Program (MVIPP) expanded in 2010, with funding from the IHS under the Tribal Injury Prevention Cooperative Agreement Program (TIPCAP), to include strategies to increase occupant protection. Funding under TIPCAP ended in August 2015. In this paper, we describe the specific program intervention components, provide data to illustrate the impact of the program 10 years after it began operation, and offer reasons for its success.

## PROGRAM IMPLEMENTATION

### Setting

The San Carlos Apache Indian Reservation is located in east-central Arizona, 110 miles east of Phoenix, Arizona. During the program period, there were an estimated 10,000 to 12,000 tribal members residing on the reservation’s 2,812 square miles. According to the 2010 census, the unemployment rate was 64%, and about 41.5% of the population lived below the poverty line.^7^ There is an IHS hospital at San Carlos, which serves primarily as an outpatient facility, and a satellite clinic about 30 miles east in Bylas. The tribal police department has its headquarters in San Carlos, with a substation located in Bylas. The number of full-time police officers has fluctuated during the past 10 years, with a low of 12 and a high of 28.

### Funding and Goals

The San Carlos MVIPP was originally funded in 2004 as one of four tribal pilot programs nationally.^8^ An MVIPP coordinator was hired in 2005. The MVIPP’s primary focus was on reducing alcohol-impaired driving,^9^ although it also conducted some media messaging regarding occupant protection. The MVIPP also creatively used incentive programs for its police officers to encourage sustained participation and motivation. The MVIPP continued to receive the CDC funding at $70,000 per year for the 5-year life of the grant, and the CDC extended the original length of the grant from 4 to 5 years due to the pilot program’s success during the initial 2 years.

Due to the demonstrated success of the MVIPP, in 2009 the San Carlos Police Department self-funded the program while it sought other funding options. The activities initiated during the 5 CDC-funded years continued during the 1 year of self-funding.

In 2010, the MVIPP received funding from TIPCAP in the amount of $65,000 per year for 5 years. The TIPCAP program had a similar intent as the earlier CDC program, namely to develop injury prevention programs based on effective strategies or best practices. As part of the TIPCAP grant, the MVIPP expanded its efforts to include strategies to increase occupant protection.

Following in approximate chronological order are the major MVIPP injury intervention components that have been accomplished from 2005 through 2013.

### Community Awareness and Outreach

The MVIPP developed a comprehensive media campaign in 2005 and 2006 that provided a foundation from which to operate for the duration of the program. Adopting a social marketing approach, the outreach made the public aware of the program and its activities. It used a variety of local media outlets to advertise its message about alcohol-impaired driving and occupant protection. These included paid advertisements in local newspapers, paid messages on the local cable access channel, messaging on the local casino marquee, and unpaid messaging via flyers provided to residents and posted on bulletin boards. Similar media outlets were used to advertise scheduled checkpoints. These culturally appropriate messages were derived from focus groups that we conducted with the public, and messages were advertised more frequently during tribal and national holidays in alignment with the Bureau of Indian Affairs Indian Highway Safety’s campaign and the National Highway Traffic Safety Administration’s campaigns.

The motor vehicle injury prevention program in San Carlos used a variety of local media outlets to spread information about alcohol-impaired driving and occupant protection.

**FIGURE 1.**
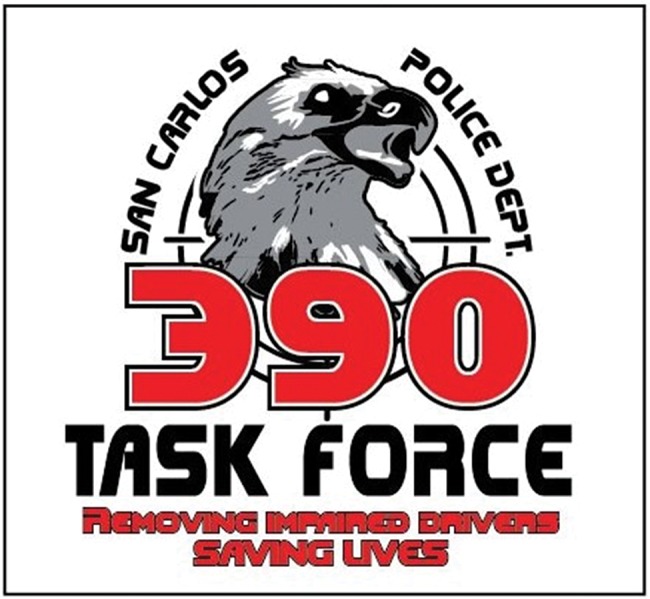
390 Task Force Logo The 390 Task Force coordinated and carried out driving under the influence (DUI) checkpoints. 390 refers to the police code for an impaired driver.

**FIGURE 2.**
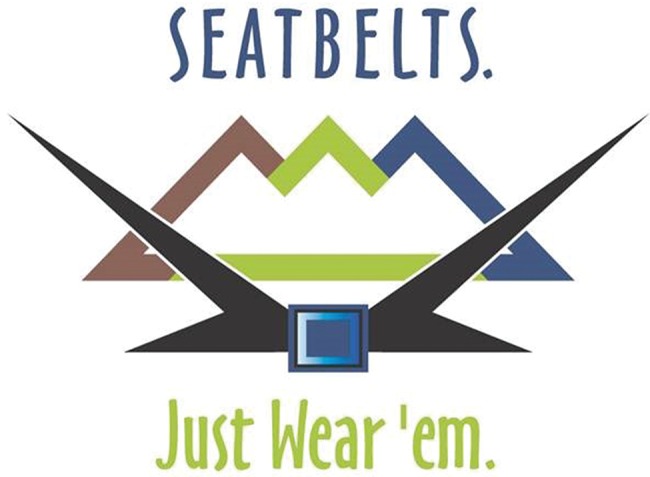
Culturally Significant Occupant Restraint Logo Triplet Mountain depicted in the logo represents a unique local landmark recognized as San Carlos specific. The incorporation of local images or artwork are often more meaningful to the local population than generic materials from other sources.

An associated media campaign activity was the development and use of specific slogans and logos associated with the MVIPP. One logo (Figure 1) referenced the 390 Task Force, which coordinated and carried out the driving under the influence (DUI) checkpoints. The term “390” refers to the police code for an impaired driver. A second logo (Figure 2) incorporated a local, culturally significant design (featuring Mount Triplet) into an occupant restraint message. Both slogans and logos were printed on a variety of promotional items valued by the public, such as t-shirts, key chains, nylon drawstring bags, and travel mugs. Third-party funding sources (e.g., Federal Highway Administration, Arizona Department of Health Services, Inter Tribal Council of Arizona) also enhanced these campaigns throughout the project.

Media messages evolved to ensure the right information reached the target audience to help promote safe behaviors. Initial “don’t drink and drive” messages were revised to “don’t drink and drive, *use a sober driver.*” Messaging was clarified because police officers discovered during the first few DUI checkpoints that non-sober drivers would state they were the least intoxicated occupant of the vehicle and were therefore designated the most likely to drive safely.

The MVIPP participated in occasional community events, including health fairs, parades, and community meetings, to advertise the program and its activities.

### DUI Enforcement

**Figure:**
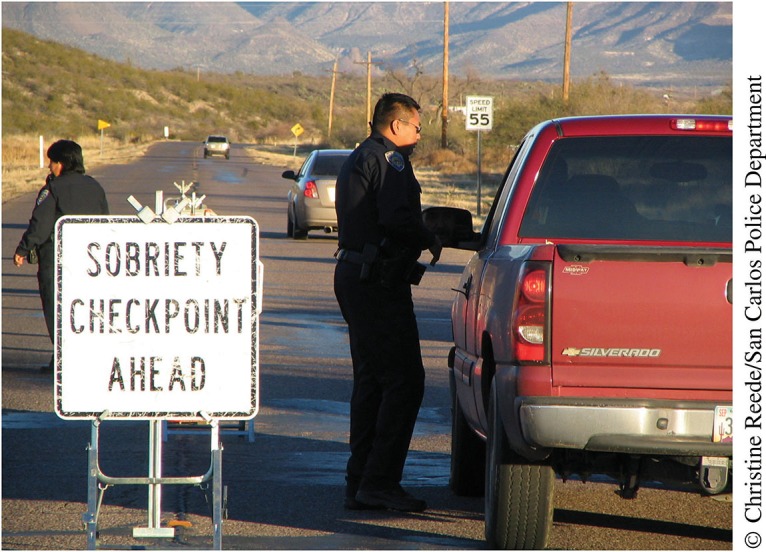
Sobriety checkpoints were an important part of the multifaceted motor vehicle injury prevention program in an American Indian Reservation in Arizona.

The MVIPP emphasis on impaired driving led to the development of a DUI task force, known as the 390 Task Force, which planned and organized the DUI checkpoints at various locations on the reservation. The high-visibility checkpoints used standard operating procedures for consistency, stopped all traffic during a specified time period to assess drivers’ levels of impairment, and involved the use of field sobriety tests and breath alcohol testers to assess alcohol impairment. The times of day and days of the week for the checkpoints were determined by anecdotal evidence contributed by the Task Force members and by police crash report data.

A task force organized regular DUI checkpoints at various locations on the reservation.

Some DUI checkpoints were conducted by San Carlos Police Department officers and support staff alone. Others were conducted with the assistance of regional enforcement partners, such as neighboring tribal police departments, neighboring city police departments, county sheriffs, the Arizona Department of Public Safety, and enforcement officers from the US Immigration and Customs Enforcement.

When police officer staffing levels or competing work priorities precluded the staff-intensive DUI checkpoints, saturation patrols were used as an alternative to checkpoints. Saturation patrols involved increased patrols at high-risk times in high-risk areas to identify impaired drivers, as well as to enforce all traffic safety laws.

### Lower BAC Limit

Lowering the legal BAC limit for vehicle drivers from 0.10% to 0.08% is a recommended intervention to reduce alcohol-impaired driving.^5^ In 2007 the San Carlos Apache Tribe lowered the legal BAC to 0.08%. The MVIPP championed this legislation and significantly contributed to this decision.

Lowering the legal blood alcohol concentration limit for drivers from 0.10% to 0.08% is recommended to reduce alcohol-impaired driving.

### Incentive Programs for Officers

The MVIPP coordinator used a variety of creative incentives—beyond overtime pay—to encourage and maintain police officers’ sustained participation and motivation in the program. Such incentives, valued by officers, included home-cooked meals before and during checkpoint events, awards of food and promotional items such as windbreakers and jackets to recognize exceptional participation and performance, an expense-paid trip to a national conference for the officer with the most DUI arrests in a calendar year, and recognition for outstanding performance in off-Reservation organizations such as Mothers Against Drunk Driving (MADD). These incentives were especially important during periods of shortages of police officers on staff.

### Primary Occupant Protection Law

In November 2011, the San Carlos Apache Tribe enacted a primary occupant restraint law, to become effective in January 2012. The tribe was the fifth Arizona tribe to pass a primary law more stringent than the state’s secondary law. With primary laws, police officers may stop vehicles solely for seat belt law violations. In contrast, with secondary laws, officers must have another reason to stop a vehicle before citing an occupant for failing to wear a seat belt.

The San Carlos Apache Tribe enacted a primary occupant restraint law, which allowed police officers to stop vehicles solely for seat belt law violations.

As part of planning the implementation of the new primary occupant restraint law, the MVIPP and San Carlos Police Department leadership realized the public needed time to adapt to the new law. Simultaneous to a media campaign, the San Carlos Police Department began a 3-month enforcement grace period, during which officers would stop drivers who were not wearing a seat belt but would only issue a warning citation. Public awareness via media outlets continued beyond the 3-month grace period.

Full enforcement of the new primary occupant restraint law began in late April 2012. At this time, anyone stopped by a police officer for not wearing a seat belt was issued a citation.

## METHODS

Evaluation of the MVIPP was an integral part of its development. Several data elements were tracked throughout the operation of the program to measure its impact and are reported here. These included DUI arrests, total San Carlos Police Department-reported crashes, nighttime crashes, crashes involving injury or fatality, and nighttime crashes involving injury or fatality. A motor vehicle crash was any vehicle incident that involved a motor vehicle occupant, pedal cyclist, pedestrian, or other transport on a public highway, street, or road. Motorcycles, all-terrain vehicles, and other off-road vehicles, were not included unless a motor vehicle was also involved in the crash.

The number of DUI arrests and the total number of crashes are self-explanatory. The injury crashes and nighttime crashes were tracked since alcohol-involved crashes are associated with greater likelihood of injury,^10^ and alcohol-involved crashes are more frequent during nighttime hours.^11^ Due to variation throughout the year for times of sunrise and sunset, and to compensate for a relatively smaller number of crashes with a shorter nighttime period, we used a standard definition for nighttime as between 6:00 pm and 5:59 am.

Joinpoint Regression Analysis Software models were used to calculate trend changes in these key data elements. Joinpoint is statistical software for the analysis of trends using joinpoint models, i.e., models where several different lines are attached at the "joinpoints." Joinpoint models linear and non-linear trend lines, which can be used to best determine significant variations in data. We report one joinpoint relevant to our DUI arrest data. For each trend segment, the annual percentage change (APC) and corresponding *P* values were calculated.

We also tracked seat belt use to measure the impact of the primary occupant restraint law, which became effective in January 2012. We observed driver and front seat passenger seat belt use according to the local IHS observational seat belt survey protocol. The protocol standardizes how surveys are conducted to ensure local seat belt use surveys are reasonably consistent and provides guidance for the selection of observation locations, survey procedures, and data summary and reporting.

In 2013, 3 police-coordinated traffic checkpoints, which stopped all traffic at a particular location, provided an opportunity to obtain an estimate of car seat use for a sample of children in the stopped vehicles. It is important to note that these observations indicated only if a child was occupying a car seat; they did not assess car seat installation or optimal restraint use (e.g., child buckled in an age- and size-appropriate car seat).

Finally, knowledge, attitudes, and practices (KAP) surveys of the public were conducted in 2005, 2006, and 2008. KAP surveys were conducted to document the level of the public's support of a lower BAC limit, a primary occupant restraint law, enhanced enforcement of new laws, and perceptions related to DUI and occupant restraints. Questionnaires were designed and piloted locally then administered by trained surveyors who completed 1 survey in 5 minutes or less. Occasionally, participant incentives such as free bottles of drinking water or t-shirts were provided. KAP survey locations were selected based on the high likelihood of being able to find a population primarily comprised of tribal members (e.g., grocery store, post office). We surveyed 200 participants in 2005, 139 in 2006, and 140 in 2008. In addition, a cost-benefit analysis of the San Carlos MVIPP, that included the program years 2005 through 2008, was conducted by a third party to estimate the value of the program’s effects.

## RESULTS

### DUI Arrests and Motor Vehicle Crashes

Table 1 shows the changes in data elements over time. The 2004 data serve as baseline measures, since the program received its initial funding in late 2004 but did not begin implementation of any program activities until early 2005.

**TABLE 1:** Number of Driving Under the Influence (DUI) Arrests and Motor Vehicle Crashes (MVC) on the San Carlos Apache Reservation, 2004 to 2013

	2004	2005	2006	2007	2008	2009	2010	2011	2012	2013	Annual Percentage Change	*P* Value
DUI arrests	308	385	411	391	468	533	359	392	375	213		
Segment 1 (2004–2009)											9.08	.12
Segment 2 (2009–2013)											-14.95	.09
Total MVCs	338	276	247	297	240	235	240	161	162	203	-6.34	.002
Nighttime^a^ MVCs	146	102	98	121	107	107	91	68	63	68	-7.43	.001
MVCs with injuries/fatalities	104	87	83	101	72	79	73	48	55	75	-5.37	.01
Nighttime^a^ MVCs with injuries/fatalities	51	33	39	39	39	46	31	22	21	26	-6.89	.02

a 6:00 pm to 5:59 am.

Overall, the change in DUI arrests was non-linear—the trend for the first segment (between 2004 and 2009) was not significant nor was the trend for the second segment (between 2009 and 2013). The model with a joinpoint at 2009 is significantly better (*P*=.02) than the linear model, implying the DUI trend is not linear. The annual percentage change (APC) increased 9.08% per year until 2009, then decreased 14.95% per year afterwards, but neither change was statistically significant (*P*=.12 and *P*=.09, respectively).

However, the remaining outcomes regarding motor vehicle crashes were linear and significant, all decreasing about 5% to 7% per year. The number of total crashes, nighttime crashes, crashes involving injury/fatality, and nighttime crashes involving injury/fatality all generally dropped by substantial proportions from 2004. The APC for total MVCs was -6.34 (*P*=.002), for nighttime MVCs -7.43 (*P*=.001), for MVCs with injuries/fatalities -5.37 (*P*=.01), and for nighttime MVCs with injuries/fatalities -6.89 (*P*=.02).

Motor vehicle crashes decreased significantly by about 5% to 7% per year.

### Occupant Restraint Use

Data on driver and front seat passenger seat belt use reflect a 144% increase, from 19% before the primary occupant restraint law was enacted (2011) to 47% during the first full year of enforcement (2013) (Table 2). While the data in Table 2 depict observed seat belt use for a short 1-year time period before the law was enacted, observed seat belt use for the years 2004 through 2010 was consistently below 20%.

Seat belt use increased from 19% in 2011 to 47% in 2013.

**TABLE 2:** Driver and Front Seat Passenger Seat Belt Use: Before and After Enactment of the Primary Occupant Restraint Law on the San Carlos Apache Reservation, 2011 to 2013

Enforcement Phase (Time Period)	N	No. Wearing Seat Belt	Percent Seat Belt Use	(95% CI)
Pre-enactment of primary seat belt law (Jan to Dec 2011)	445	86	19.3%	(15.7, 23.0)
Enforcement grace period^a^ (Feb to Apr 2012)	237	72	30.4%	(24.5, 36.2)
Full enforcement of primary seat belt law (May to Dec 2012)	822	354	43.1%	(39.7, 46.5)
Full enforcement of primary seat belt law (Jan to Dec 2013)	851	400	47.0%	(43.7, 50.4)

a Police officers stopped vehicles if occupants were not restrained but only issued warning citations.

The tribe’s primary occupant restraint law also encompasses child car seat use. Efforts to assess the extent of car seat use prior to 2013 varied by methodology and venue. Many of these assessments involved voluntary car seat distribution or check events, which did not provide representative estimates of the population but found car seat use at less than 20%. In 2013, 3 police-coordinated traffic checkpoints that stopped all traffic at a particular location provided an opportunity to obtain a better estimate of car seat use for a sample of children in the stopped vehicles. In 2013, the combined observations from 3 checkpoints found that 52% of the children were in car seats. If we use 20% car seat use from previous years as the baseline measure, this reflects a 160% increase in car seat use since the occupant restraint law was enacted.

**Figure:**
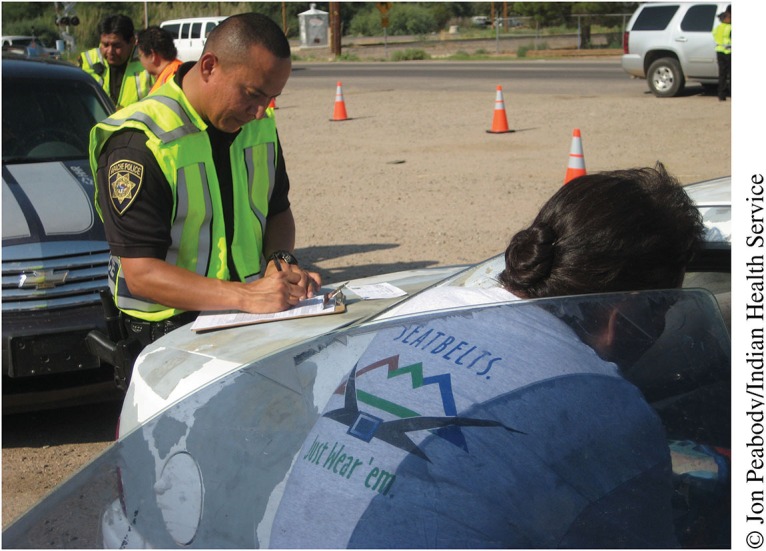
A certified child passenger safety technician and police officer provide education and help parents install a new car seat at a car seat distribution and check event.

### Public Attitudes About Motor Vehicle Injury Prevention

KAP surveys revealed high levels of support for programs to prevent DUI. For example, in 2005 81% of KAP survey respondents were in favor of sobriety checkpoints, increasing to 93% in the 2008 KAP survey (Table 3). Nearly all (95%) survey respondents in 2006 thought there should be a child car seat safety law. Over time, more people reported seeing or being stopped at a DUI checkpoint in the past 12 months (increasing between 2005 and 2008 from 42% to 71% for seeing a checkpoint, and from 31% to 54% for being stopped at a checkpoint). Knowledge of appropriate designated drivers also increased, from 70% in 2005 stating a designated driver should have no drinks before driving to 86% in 2008.

**TABLE 3:** Results From Knowledge, Attitudes, and Practices (KAP) Surveys of the Public, Motor Vehicle Injury Prevention Program, San Carlos Apache Reservation, 2005, 2006, 2008

KAP Survey Variable	2005 (N = 200)	2006 (N = 139)	2008 (N = 140)
In favor of sobriety checkpoints	81%	NA	93%
Indicated it was “very important” to do something to reduce drinking and driving	94%	NA	97%
Reported seeing a DUI checkpoint in operation in the past 12 months	42%	NA	71%
Reported being stopped at a DUI checkpoint in the past 12 months	31%	NA	54%
Reported seeing or hearing a message in the media about drunk driving in the past 12 months	62%	NA	73%
Stated a designated driver should have no drinks before driving	70%	NA	86%
Thought there should be a law requiring all children to ride in a car seat	NA	95%	NA

Abbreviation: DUI, driving under the influence.

### Cost-Benefit Analysis

The cost-benefit conducted by an independent third party of program years 2005 through 2008 concluded that the MVIPP showed a lifetime cost-benefit ratio of 1:9.86, meaning that for every US$1 dollar spent to implement the program, there was almost $10 in savings from reduced medical and other costs.^12^

## DISCUSSION

The San Carlos MVIPP clearly demonstrates the application of evidence-based injury prevention strategies is feasible and has a positive impact on public health in a Native American community. Use of such effective strategies as instituting DUI checkpoints to enforce sobriety laws, lowering the legal BAC limit for drivers, and passing and enforcing primary occupant restraint laws significantly reduced motor vehicle crashes, including those involving injuries or fatalities, and increased occupant restraint use, including car seat use. In 2013, the data on motor vehicle crashes suggest an upward turn, which may be due to reduced police officer staffing at the San Carlos Police Department.

Changes in DUI arrests over time were not statistically significant. However, the number of DUI arrests generally increased over the first several years of the project, but then the number declined or plateaued over the remaining project period. Possible explanations for this decline include that the San Carlos Police Department had significantly fewer sworn officers or that officers had multiple responsibilities other than traffic enforcement that competed with, and often negated, opportunities for involvement in DUI and/or occupant protection checkpoints. On the other hand, the decline in DUI arrests could be related to increased awareness among community members about the danger of DUI or the risk of DUI arrest leading to less impaired driving incidents.

We believe several factors contributed to the program’s overall success:

- Extensive partnerships
- Technical expertise
- Program managed by a civilian employee within the police department
- Consistent staffing and funding
- DUI task force
- Use of incentives
- Community and stakeholder support

Although these factors are valuable assets individually, all of them combined likely led to the program’s significant success. Partners included federal agencies (e.g., Indian Health Service, the CDC, Bureau of Indian Affairs), multiple law enforcement agencies (tribal, city, county, state, federal), and a private marketing firm. A high level of local technical expertise existed. The public health staff involved with the program contributed expertise in the collection of qualitative and quantitative data, and in the management, analysis, interpretation, and presentation of that data. The law enforcement staff involved with the program contributed expertise in the development, enactment, and implementation of laws; with the enforcement of laws in general; and with specific enforcement operations such as DUI checkpoints.

The establishment of the MVIPP was the result of proactive foresight from the police department to become involved in a “prevention” effort. This highlights the point that not all prevention programs should or must be managed by a public health program to be successful. The department also hired a civilian (i.e., non-officer) full-time program coordinator who was able to dedicate all her talent and resources to implementing the interventions. Both the department and the coordinator possessed unwavering dedication to the project and to achieving its goals from the program’s inception.

The creation of a DUI task force within the police department emphasizes the department’s dedication to the issue. Reliable financial resources, through the multiyear funding cycles, enabled the program to function at a level necessary to implement the effective strategies. This included money for police officer overtime pay and the use of funding sources outside the department to procure equipment used at sobriety checkpoints.

Many of these factors are consistent with what Fell et al.^13^ identified as reasons for enforcement agencies to conduct sobriety checkpoints, including active local task forces who manage checkpoints, available financial and human resources whether from within law enforcement agencies or from outside sources, and support from the general public and officials to deter alcohol-impaired driving. The formation of impaired driving task forces or coalitions and support from local organizations for high-visibility law enforcement efforts, such as sobriety checkpoints, have been recommended elsewhere as well.^14^^,^^15^ We believe in addition to these elements, a major factor that promoted involvement in the checkpoints was the incentive program used to encourage and reward police officers of the DUI task force. Incentives included overtime pay, meals, and socializing among the task force prior to conducting sobriety checkpoints.

The support from tribal and police leadership was critical throughout the life of the program. Such ardent support was evident in the passage of the 0.08% BAC law and the primary occupant restraint law as well as the enforcement of these new laws. Support was also manifested in less obvious ways, such as police officer participation in injury prevention-focused training courses, conferences, and program activities such as DUI and occupant restraint checkpoints. We believe these interventions worked because they were championed by local leaders and because law enforcement and public health officials alike shared the common goal of reducing motor vehicle-related injuries, encouraging close collaboration on achieving this goal throughout the program period.

Support from tribal and police leadership was critical to the success of the injury prevention program.

A novel element of the program’s data and monitoring strategy was not only to provide monitoring and evaluation updates to the funders but also to document the program’s approaches and share program successes and challenges with the broader public health community. For example, the program developed and presented several posters at the National Conference on Highway Safety Priorities – Lifesavers on topics ranging from overall program evaluation to unique program elements such as the police officer incentive program.^16^^-^^23^ We have found that such knowledge sharing activities offered several unexpected benefits (Box), and we recommend multifaceted community-based programs to consider incorporating such activities into their program evaluation.

**BOX. Benefits of the Motor Vehicle Injury Prevention Program Sharing Successes and Challenges With Broader Public Health Community**

- **Ensures data collection/analysis.** Effective data management may be incomplete in under-resourced programs.
- **Develops new skills.** Improves written and oral communication skills, which provides long-term benefits for team members to better explain their mission and how they plan to achieve goals and overcome barriers.
- **Reports back to stakeholders.** Demonstrates program’s initiatives, successes, and challenges in different formats for local stakeholders/partners and for tribal leadership.
- **Increases exposure.** Allows local practitioners the opportunity to interact with highway safety and injury prevention colleagues from across the country.
- **Enhances morale.** Enhances sense of accomplishment and motivates partners, particularly when work such as conference posters is displayed in routine workspaces such as police officer patrol rooms or local hospital/clinic corridors, as well as markets the program to the public and to funding sources.

### Strengths and Limitations

A challenge that was overcome early in the program was the collection and management of data. Since the program coordinator received training in injury prevention and data management, she became the on-site data advocate within the police department. She worked closely with the officers to stress the importance of accurate and complete crash reports, and to emphasize the importance of the data they collect in measuring the impact of the MVIPP. She, in turn, collected and managed the collective crash data. This early emphasis on quality data resulted in the data component becoming a strength of the MVIPP. The data were used to demonstrate the program’s impact on several platforms: as feedback to the officers to reinforce the importance of their enforcement and data collection efforts, to police management and tribal leadership to demonstrate the value of the program in preventing injury, to the funding agencies to demonstrate the value of their investments, and to procure additional funds from multiple sources.

Data variables are police-reported only, with no verification by health care sources. While such verification through health care sources may be ideal, it was beyond the scope of local resources to combine with local emergency room data. This was recognized in a 2010 report that surprisingly determined police-reported MVCs were a more reliable data source than accessible local health care facility data sources because many MVC victims were evacuated directly from the crash site to health care facilities that provided a higher level of care.^24^

A decrease in DUI arrests and increases in the 4 crash variables in 2013 (Table 1) coincided with a very low San Carlos Police Department officer staffing level. While we believe the low officer staffing likely contributed to this 2013 data pattern, we cannot conclusively establish a causative effect. Regardless, a challenge for a small police department without a dedicated traffic enforcement unit is keeping focus on traffic enforcement during times of reduced police officer staffing, with competing law enforcement issues similarly requiring their attention.

Program sustainability beyond the time period we report in this paper is difficult to predict. However, continued collaboration among partners is likely and regardless of level of funding, interventions conducted may be scaled to meet local needs based on available resources (e.g., fewer DUI checkpoints, or low-manpower checkpoints, and increased saturation patrols).

## CONCLUSION

The San Carlos Apache Tribe’s Motor Vehicle Injury Prevention Program is an exceptional example that supports the "Community Guide’s" recommendations that a carefully planned, well-executed, multicomponent program, implemented in conjunction with community mobilization efforts, can reduce motor vehicle crashes and increase occupant restraint use. Key factors that contributed to the program’s success include strong partnerships between public health and law enforcement agencies, consistent financial resources over multiple years, and high-visibility enforcement of impaired driving laws.

## Notes

**Peer Reviewed**
